# Canine Respiratory Coronavirus, Bovine Coronavirus, and Human Coronavirus OC43: Receptors and Attachment Factors

**DOI:** 10.3390/v11040328

**Published:** 2019-04-05

**Authors:** Artur Szczepanski, Katarzyna Owczarek, Monika Bzowska, Katarzyna Gula, Inga Drebot, Marek Ochman, Beata Maksym, Zenon Rajfur, Judy A Mitchell, Krzysztof Pyrc

**Affiliations:** 1 Microbiology Department, Faculty of Biochemistry, Biophysics and Biotechnology, Jagiellonian University, Gronostajowa 7, 30-387 Krakow, Poland; artur.szczepanski@doctoral.uj.edu.pl (A.S.); katarzyna.kosowicz@uj.edu.pl (K.O.); 2 Virogenetics Laboratory of Virology, Malopolska Centre of Biotechnology, Jagiellonian University, Gronostajowa 7a, 30-387 Krakow, Poland; katarzyna.gula@uj.edu.pl (K.G.); inga.drebot@uj.edu.pl (I.D.); 3 Department of Cell Biochemistry, Faculty of Biochemistry, Biophysics and Biotechnology, Jagiellonian University, Gronostajowa 7, 30-387 Krakow, Poland; monika.bzowska@uj.edu.pl; 4 Department of Cardiac, Vascular and Endovascular Surgery and Transplantology, Medical University of Silesia in Katowice, Silesian Centre for Heart Diseases, Marii Curie Sklodowskiej 9, 41-800 Zabrze, Poland; M.Ochman@sccs.pl; 5 Department of Pharmacology, School of Medicine with the Division of Dentistry in Zabrze, Medical University of Silesia in Katowice, ul. Jordana 19, 41-808 Zabrze, Poland; Bea2104@interia.pl,; 6 Institute of Physics, Faculty of Physics, Astronomy and Applied Computer Sciences, Jagiellonian University, Lojasiewicza 11, 30-348 Krakow, Poland; zenon.rajfur@uj.edu.pl; 7 Department of Pathology and Pathogen Biology, The Royal Veterinary College, Hatfield, Hertfordshire AL9 7TA, UK; jmitchell@rvc.ac.uk

**Keywords:** canine respiratory coronavirus, bovine coronavirus, human coronavirus OC43, attachment receptor, entry receptor, sialic acids, sialic acids, coronavirus, entry, HLA

## Abstract

Despite high similarity of canine respiratory coronavirus (CRCoV), bovine coronavirus, (BCoV) and human coronavirus OC43 (HCoV-OC43), these viruses differ in species specificity. For years it was believed that they share receptor specificity, utilizing sialic acids for cell surface attachment, internalization, and entry. Interestingly, careful literature analysis shows that viruses indeed bind to the cell surface via sialic acids, but there is no solid data that these moieties mediate virus entry. In our study, using a number of techniques, we showed that all three viruses are indeed able to bind to sialic acids to a different extent, but these molecules render the cells permissive only for the clinical strain of HCoV-OC43, while for others they serve only as attachment receptors. CRCoV and BCoV appear to employ human leukocyte antigen class I (HLA-1) as the entry receptor. Furthermore, we identified heparan sulfate as an alternative attachment factor, but this may be related to the cell culture adaptation, as in ex vivo conditions, it does not seem to play a significant role. Summarizing, we delineated early events during CRCoV, BCoV, and HCoV-OC43 entry and systematically studied the attachment and entry receptor utilized by these viruses.

## 1. Introduction

Coronaviruses are enveloped, single-stranded, positive-sense RNA viruses that cluster within the family *Coronaviridae* (order *Nidovirales*). They infect a wide variety of species, including humans, livestock, and companion animals. In humans, they usually cause mild to moderate respiratory symptoms, although more severe cases occur mostly in individuals with impaired immune responses [1]. The emergence of severe acute respiratory syndrome coronavirus (SARS-CoV) in China in the winter of 2002, and of Middle East Respiratory Syndrome coronavirus (MERS-CoV) in September 2012, reminds us that coronaviruses pose a significant threat, and that zoonotic transmission is common [2,3,4,5,6].

**Figure 1:**
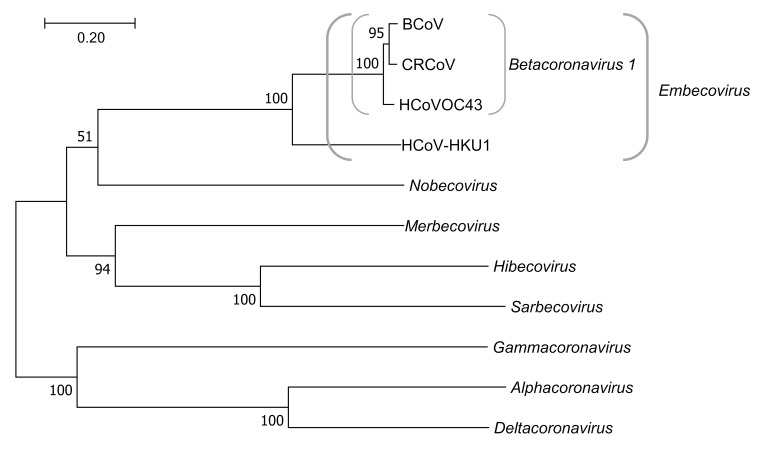
Phylogenetic tree of *Orthocoronavirinae*. The evolutionary history was inferred based on the sequences of the complete spike gene by using the Maximum Likelihood method based on General Time Reversible model [7]. The tree with the highest log likelihood-33860.47) is shown. The percentage of trees in which the associated taxa clustered together is shown next to the branches. Initial tree(s) for the heuristic search were obtained automatically by applying Neighbor-Join and BioNJ algorithms to a matrix of pairwise distances estimated using the Maximum Composite Likelihood (MCL) approach, and then selecting the topology with superior log likelihood value. Discrete Gamma distribution was used to model evolutionary rate differences among sites (5 categories (+G, parameter = 3.0783)). The rate variation model allowed for some sites to be evolutionarily invariable ([+I], 5.04% sites). The tree is drawn to scale, with branch lengths measured in the number of substitutions per site. The analysis involved 11 nucleotide sequences. All positions containing gaps and missing data were eliminated. There was a total of 3117 positions in the final dataset. Evolutionary analyses were conducted in MEGA7 [8]. *Alphacoronavirus*: Human coronavirus 229E (HCoV-229E; NC_002645); *Deltacoronavirus*: Wigeon coronavirus HKU20 (NC_016995); *Gammacoronavirus*: Beluga Whale coronavirus SW1 (NC_010646); BCoV: Bovine coronavirus (NC_003045); CRCoV: Canine respiratory coronavirus (JX860640); HCoV-OC43: Human coronavirus OC43 (NC_006213); HCoV-HKU1: Human coronavirus HKU1 (NC_006577); *Hibecovirus*: Bat Hp-betacoronavirus/Zhejiang2013 (NC_025217); *Merbecovirus*: Middle East respiratory syndrome coronavirus (NC_019843); *Nobecovirus*: Bat coronavirus HKU9 (NC_009021); *Sarbecovirus*: SARS coronavirus (NC_004718).

The *Coronaviridae* family is divided into two subfamilies, *Letovirinae* and *Orthocoronavirinae*, and the latter one is further divided into four genera—alpha, beta, gamma, and delta—based on the degree of nucleotide similarity. The current phylogeny of the family is presented in Figure 1.

Betacoronaviruses are highly variable and use a number of different attachment and entry receptors. Generally, it is believed that viruses from this genus attach to the glycan layer on the cell surface, (e.g., sialic acids (SAs) or heparan sulfate (HS)) [9,10,11,12,13,14]. The second step during the infection is interaction with the entry receptor. Several molecules were proposed to serve as such, including carcinoembryonic antigen-cell adhesion molecule (CEACAM1) for murine hepatitis virus (MHV), angiotensin-converting enzyme 2 (ACE2) for SARS-CoV and dipeptidyl peptidase 4 (DPP4) for MERS-CoV. Human leukocyte antigen class I (HLA-I) was suggested to serve as entry receptor for HCoV-OC43 [15,16] and HCoV-HKU1 [17], but these discoveries were not confirmed in subsequent studies. Interestingly, SAs were also reported to serve as entry receptors, but some speculate that they play a negligible role in cell entry [15,16,17].

HCoV-OC43 was first isolated in 1967. It is (next to HCoV-NL63) the most common coronavirus in humans. Incidence of infection is the highest in winter and early spring [18,19,20]. It is thought that the virus emerged in the human population at the end of the 19th century, but the most recent common ancestor has not been identified. Of interest, a number of betacoronaviruses (e.g., bovine coronavirus (BCoV), canine coronavirus (CRCoV), and dromedary camel coronaviruses) are closely related to this human pathogen; it was suggested that these species recombine and new subspecies emerge [19,21,22,23].

Here, we focused on three closely related betacoronaviruses; they are almost identical from a genetic perspective but cause disease in different hosts. HCoV-OC43 causes a common cold in humans, BCoV causes gastrointestinal and respiratory tract disease in cattle [24,25,26], and CRCoV is linked to kennel cough in dogs. Surprisingly high resemblance of their spike proteins (97.3% nucleotide identity for CRCoV and BCoV and 96.9% for CRCoV and HCoV-OC43 [27]) not common among betacoronaviruses [23], as well as cross-reactivity of BCoV antigen with canine antibodies [27] and infectivity of BCoV to pups [22,28] led us to ask whether these three viruses share receptor specificity. We found that both SAs and HS serve as attachment factors for all three viruses, albeit to different extent. However, SAs are not essential for CRCoV and BCoV infection and serve as entry receptors only for clinical strain of HCoV-OC43, whereas CRCoV and BCoV employ HLA-I.

## 2. Materials and Methods

### 2.1. Cells

HRT-18G (ATCC CRL-11663) cells, derivative of HRT-18 (ATCC CCL-244, ileocecal colorectal adenocarcinoma) were maintained in Dulbecco’s MEM (Life Technologies, Warsaw, Poland) supplemented with 3% heat-inactivated fetal bovine serum (Life Technologies, Warsaw, Poland), penicillin (100 U/mL), streptomycin (100 μg/mL), and ciprofloxacin (5 μg/mL). Cells were cultured at 37 °C under 5% CO_2_.

To generate human airway epithelium (HAE) cultures, primary cells isolated from patients’ tissue were cultured on plastic in Bronchial Epithelial Growth Media (BEGM) medium for 1–2 weeks. Next, passage 2 cells were seeded on permeable Transwell insert supports (12 mm) and after the confluence was reached, apical medium was removed, and cells were grown for another 5–8 weeks on air–liquid interface (ALI) in ALI medium. After this time, cells were fully differentiated, forming pseudostratified mucociliary epithelium.

### 2.2. Viral Stocks

HCoV-OC43, CRCoV, and BCoV stocks were prepared by infecting HRT-18G cells. HCoV-OC43 (ATCC: VR-1558) was cultured in Dulbecco’s MEM (Life Technologies, Warsaw, Poland) supplemented with 2% heat-inactivated fetal bovine serum (Life Technologies, Warsaw, Poland), penicillin (100 U/mL) and streptomycin (100 μg/mL) at 32 °C under 5% CO_2_. BCoV Mebus strain (NR-445, BEI Resources) was cultured in Dulbecco’s MEM (Life Technologies, Warsaw, Poland) supplemented with penicillin (100 U/mL) streptomycin (100 μg/mL) and trypsin (1 µg/mL) at 37 °C under 5% CO_2_. CRCoV strain 4182 was cultured in Dulbecco’s MEM (Life Technologies, Warsaw, Poland) supplemented with penicillin (100 U/mL) and streptomycin (100 μg/mL) at 37 °C under 5% CO_2_. Stocks were collected at 5th day post infection (p.i.) by two freeze-thaw cycles. Mock-infected cells were used as a control. Virus’ yield was estimated by titration according to the Reed and Muench formula [29]. Obtained aliquots were stored at −80 °C.

HCoV-NL63 stock (isolate Amsterdam 1) was generated by infecting LLC-MK2 cells cultured in MEM with Hanks’ and Earle’s salts (two parts Hanks’ MEM and one part Earle’s MEM) supplemented with 3% heat-inactivated fetal bovine serum (Life Technologies, Warsaw, Poland), penicillin (100 U/mL) and streptomycin (100 μg/mL) at 32 °C under 5% CO_2_. Stocks were collected at day 5 p.i. by two freeze-thaw cycles. Mock-infected cells were used as a control.

Influenza A virus Kilbourne F108: A/Aichi/2/1968 (HA, NA) x A/Puerto Rico/8/1934 (H3N2), Reassortant X-31 was generated by infecting MDCK cells cultured in Dulbecco’s MEM (Life Technologies, Warsaw, Poland) supplemented with penicillin (100 U/mL) streptomycin (100 μg/mL) and trypsin (1 µg/mL) at 37 °C under 5% CO_2_. Stocks were collected at day 2 p.i. by two freeze-thaw cycles. Mock-infected cells were used as a control. HCoV-OC43 isolate 0500 was propagated on fully differentiated HAE cultures. Prior to the infection, the apical surfaces of HAE were washed thrice with PBS and inoculated with 100 μL of 1000× diluted viral stock [30]. After 3 h incubation at 32 °C, the unbound viral particles were removed by 3 washes with 500 μL of PBS for 5 min at 32 °C. HAE cultures were further maintained at 32 °C for 2–5 days. The new viral stock was obtained by washing of the apical surface with PBS.

### 2.3. Purification of CRCoV

Virus-containing medium was concentrated using Amicon Ultra, 10 kDa cut-off (Merck, Warsaw, Poland) and subsequently overlaid on 15% iodixanol solution in PBS (OptiPrep medium; Sigma-Aldrich, Poznan, Poland) and centrifuged at 45,000× *g* for 3 h at 4 °C. Following centrifugation virus-containing fraction was overlaid on 10–20% iodixanol gradient and centrifuged at 45,000× *g* for 18 h at 4 °C. Fractions collected from gradient were analyzed by Western blotting to detect the CRCoV nucleocapsid protein. The virus-enriched fractions were aliquoted and stored at −80 °C.

### 2.4. Antibodies to CRCoV N Protein

CRCoV nucleocapsid gene was codon optimized, synthetized, and cloned into pETDuet vector via BamHI and AatII sites. Plasmid identity was confirmed by sequencing. N protein was expressed in *E. coli* Origami cells following induction with 0.5 mM isopropyl-β-d-thiogalactopyranoside (IPTG) at 20 °C for 16 h. Protein was purified on Ni-nitrilotriacetic acid resin (IMAC Sepharose 6 Fast Flow).

Mouse polyclonal antibodies specific to CRCoV N protein were developed according to the standard protocol [31]. Six-week-old Balb/C mice purchased from The Mossakowski Medical Research Centre, Polish Academy of Sciences, Warsaw, Poland. Mice were injected intraperitoneally with 100 µg of purified recombinant protein diluted in PBS and mixed 1:1 with Complete Freund’s Adjuvant (Sigma-Aldrich, Poznan, Poland). Subsequent animal immunizations were performed weekly with 50 µg of antigen mixed with Incomplete Freund’s Adjuvant (Sigma-Aldrich, Poznan, Poland). As soon as the anti-N serum titer reached 1:1,000,000 in ELISA, animals were euthanized and blood was collected by cardiac puncture. Antibodies were purified from serum by affinity chromatography on Capture Select LC-kappa (mur) Affinity Matrix according to the manufacturer’s instructions (Thermo Fisher Scientific, Warsaw, Poland) and then dialyzed into sterile PBS.

### 2.5. Western Blot Analysis

Cell lysates were mixed with sample buffer (0.5 M Tris, pH 6.8, 10% SDS, 50 mg/mL dithiothreitol [DTT]) and boiled for 5 min. Afterwards, they were separated by SDS-PAGE electrophoresis alongside PageRuler prestained protein size markers (Thermo Fisher Scientific, Warsaw, Poland) and electrotransferred onto activated PVDF membrane. The membranes were then blocked overnight (4 °C) with 5% skim milk in Tris-buffered saline supplemented with 0.5% Tween 20. An anti-CRCoV N protein antibodies (1:200, 2 h) followed by horseradish peroxidase labeled rabbit antimouse secondary antibodies (1:20,000, 1 h, Dako, Swarzewo, Poland) were used for detection of virus. The signal was developed using Immobilon Western Chemiluminescent HRP Substrate (Millipore, Warsaw, Poland).

### 2.6. Hemagglutination Assay

Hemagglutination assay was performed in V-shaped 96-well plates. Mouse erythrocyte suspension was prepared by three washes of twelve-week-old mouse blood with PBS. The obtained 3% mouse erythrocyte suspension was mixed with viral stocks and incubated at room temperature for 1 h until developed. As controls served influenza A H3N2 reported to hemagglutinate erythrocytes [32] and HCoV-NL63 which does not bind to SAs and therefore does not trigger such effect [33]. Pictures were acquired using CANON EOS 40D camera. All viral stocks (except for influenza A) were used at TCID_50_ = 500,000. Influenza A virus was applied at TCID_50_ = 30,000, which was the highest possible titer to be obtained.

### 2.7. Virus Attachment

To determine which cell surface molecules are responsible for virus’ attachment, competition experiments were designed, or SAs residues were enzymatically removed from the surface of cells. Cells were grown on coverslips for 48 h, washed twice with PBS and incubated for 30 min at 37 °C with type II neuraminidase (NA, 100–200 mU/mL) from *Vibrio cholerae* (N6514, Sigma-Aldrich, Poznan, Poland), *N*-acetylneuraminic acid (Neu5Ac 40–80 mM, A0812 Sigma-Aldrich, Poznan, Poland), heparan sulfate (HS, 10–100 µg/mL), d-(+)-galactose (Gal, 50 mM), d-(+)-glucose (Glu, 50 mM), d-(+)-mannose (Man, 50 mM), or *N*-acetyl-d-glucosamine (NAc, 50 mM) in PBS. Following incubation, cells were cooled, washed thrice with ice-cold PBS and overlaid with viral stocks. In case of Neu5Ac virus was additionally preincubated with the compound for 1 h at 4 °C before adsorption. Sugar moieties, Neu5Ac and HS but not NA were present during adsorption. Cells were incubated for 2 h at 4 °C, washed twice with ice-cold PBS and fixed with 4% formaldehyde. Analysis was carried out with flow cytometry or confocal microscopy.

### 2.8. Confocal Microscopy

Fixed cells were permeabilized using 0.5% Tween-20 (RT, 10 min, Bioshop) and unspecific binding sites were blocked using 5% bovine serum albumin in PBS (4 °C, overnight) prior to staining. For visualization of viruses, anticoronavirus antibody OC43 strain (1 µg/mL, 2 h, RT, Merck, Warsaw, Poland) coupled with goat antimouse Alexa Fluor 488 antibody (5 µg/mL, 1 h, RT, Thermo Fisher Scientific, Warsaw, Poland) were used. After incubation with antibodies, cells were washed thrice with 0.5% Tween-20 in PBS. Nuclear DNA was stained with 4′,6′-diamidino-2-phenylindole (DAPI, 0.1 μg/mL, Sigma-Aldrich, Poznan, Poland). Stained coverslips were mounted on glass slides in Prolong Diamond medium (Thermo Fisher Scientific, Warsaw, Poland). Fluorescent images were acquired using Zeiss LSM 710 confocal microscope (Carl Zeiss Microscopy GmbH, Poznan, Poland).

### 2.9. Flow Cytometry

For flow cytometry analysis, cells were permeabilized (0.5% Tween-20 in PBS, 20 min RT) and blocked (5% bovine serum albumin in PBS, 2 h RT) prior to staining. Viruses were visualized using anticoronavirus antibody OC43 strain (1 µg/mL, 2 h, RT, Merck, Warsaw, Poland) coupled with goat antimouse Alexa Fluor 488 antibody (5 µg/mL, 1 h, RT, Thermo Fisher Scientific, Warsaw, Poland). Stained cells were scratched off the glass in PBS prior to analysis. A minimum of 10,000 cells per sample were analyzed. Cells were gated as shown in Figure S1.

### 2.10. Virus Replication

To determine whether certain proteins or sugar moieties may serve as entry receptors, cell monolayers were washed with PBS and incubated with one of the following compounds: NA (200 mU/mL), HS (100–600 µg/mL), Neu5Ac (5–80 mM), Gal (100 mM), Glu (100 mM), Man (100 mM), NAc (100 mM), polyclonal rabbit anti-HLA A/B/C antibodies (0.25–4 µg/mL, sc-30204, Santa Cruz) for 60 min at 37 °C. After three washes (with exception to HS treated cells), cells were infected with the viruses at TCID_50_ of 400 per milliliter (M.O.I. = 0.0007) in the presence of tested agent or control media. NA treatment was repeated every 24 h until the day of sample collection. After 2 h, unbound virions were washed off and the cells were further cultured in the presence of compounds at conditions appropriate for each virus (see *Viral stocks* section for details). Antibody specificity was confirmed with isotype control antibodies used at the same concentrations.

### 2.11. Reverse Transcription Quantitative PCR (RT-qPCR)

At day 5 p.i., supernatants were collected and total RNA was isolated using Viral DNA/RNA Kit (A&A Biotechnology, Gdynia, Poland). This time point was selected based on replication curves for all three viruses (Figure S2). Reverse transcription was carried out with High Capacity cDNA Reverse Transcription Kit (Thermo Fisher Scientific, Warsaw, Poland), according to the manufacturer’s protocol. Serial dilutions of quantified DNA fragment corresponding to the viral N gene cloned with InsTAclone PCR cloning kit (Thermo Fisher Scientific, Warsaw, Poland) into pTZ57R/T plasmid were used as standards. Concentration of the linearized form of the plasmid was assessed using a spectrophotometer and its purity was confirmed by gel electrophoresis.

Subsequently, PCR was performed with using KAPA PROBE FAST qPCR Master Mix (Kapa Biosystem). Rox was used as a reference dye. The amplification program, specific probes and primers for each virus are listed in Table 1.

### 2.12. Resialylation

To test whether recovery of the SAs after neuraminidase-mediated desialylation affects the virus attachment, appropriate experiments were designed. Cells grown on coverslips were washed twice with PBS, incubated for 30 min at 37 °C with 500 mU/mL NA. Subsequently cell cultures were washed with PBS and treated with 1 mM cytidine-5′-monophospho-*N*-acetylneuraminic acid (CMP-Neu5Ac, C8271 Sigma-Aldrich, Poznan, Poland) and either α-2,3-sialyltransferase (α2,3-ST) from *Pasteurella multocida* (S1951, Sigma-Aldrich, Poznan, Poland) or α-2,6-sialyltransferase (α2,6-ST) from *Photobacterium damsel* (S2076, Sigma-Aldrich, Poznan, Poland) at varying concentrations for 2 h at 37 °C. Following treatment, cells were washed thrice with ice-cold PBS and infected with iodixanol concentrated CRCoV, BCoV orHCoV-OC43. After 2 h at 4 °C, unbound virions were washed off with PBS and cells were fixed with 4% formaldehyde. Activity of neuraminidase and sialyltransferases was verified with α-2,6-SAs specific fluorescein labeled *Maackia Amurensis* lectin I (Vector Labs) and α-2,3-SAs specific Cy3 labeled *Sambucus Nigra* lectin (Vector Labs)

### 2.13. Data Analysis

All experiments were performed at least three times in triplicate. All graphs presented in this work were created using GraphPad Prism 6 software. Distribution of values was tested using Shapiro–Wilk normality test and equality of group variances was examined with Browne–Forsythe test. Depending on the results of these tests, one-way ANOVA with Dunnett’s multiple comparisons test or Kruskal–Wallis test with Dunn’s multiple comparisons test or unpaired *t* test with Welch’s correction were used. *p* values < 0.05 were considered significant. One asterisk (*) identifies adjusted *p* values between 0.01 and 0.05, two asterisks (**) identify adjusted *p* values between 0.01 and 0.001, three asterisks (***) identify adjusted *p* values between 0.001 and 0.0001, four asterisks (****) identify adjusted *p* values below 0.0001. Images obtained from the confocal microscope were deconvolved using AutoQuant X3 software and processed in ImageJ Fiji [34]. Data obtained from FACS analysis were collected using BD Cell Quest Pro software and analyzed using FlowJo V10.

### 2.14. Ethics Statement

The HRT-18G cell line and canine respiratory coronavirus (CRCoV) strain 4182 were provided by Judy A. Mitchell. BCoV, Mebus, NR-445 was obtained through the NIH Biodefense and Emerging Infections Research Resources Repository, NIAID. Influenza virus Kilbourne F108: A/Aichi/2/1968 (HA, NA) x A/Puerto Rico/8/1934 (H3N2), Reassortant X-31 (Derived from Mouse-adapted X-31b), NR-3483 was obtained through the NIH Biodefense and Emerging Infections Research Resources Repository, NIAID. Human coronavirus OC43 (HCoV-OC43) VR-1558 and MDCK cell line (ATCC CCL-34) were obtained from American Type Culture Collection (ATCC). LLC-MK2 cells were kindly provided by Lia van der Hoek. HCoV-NL63 (isolate Amsterdam 1) was obtained as described [35]. The authors are grateful to Volker Thiel for providing HCoV-OC43 isolate 0500.

HAE cells were obtained from airway specimens resected from patients of Silesian Center for Heart Diseases. Following written informed consent of all subjects to participate in this study, the procedure of acquisition was carried out according to the protocol approved by the Bioethical Committee of the Medical University of Silesia in Katowice, Poland (approval no: KNW/0022/KB1/17/10 dated on 16 February 2010).

All animal procedures were in agreement with the guidelines of the Institutional Animal Care and Use Committee (IACUC) and the whole study was approved by 2nd Local IACUC in Kraków (Institute of Pharmacology Polish Academy of Sciences). Further, all animal procedures complied with the Act on the Protection of Animals used for scientific or educational purposes dated on 15th of January 2015 (D20150266L), which implements the Directive of the European Parliament and the Council (2010/63/EU) dated on 22nd of September 2010.

## 3. Results

### 3.1. HCoV-OC43, BCoV, and CRCoV Use SAs to Attach to Target Cells

**Figure 2:**
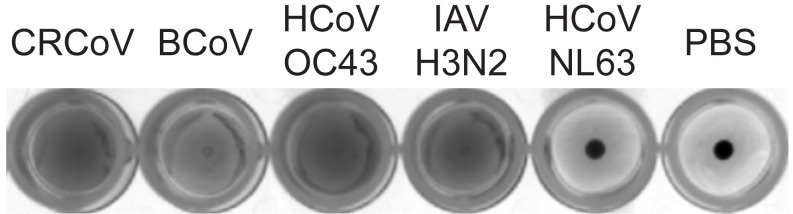
HCoV-OC43 VR-1558, BCoV and CRCoV agglutinate erythrocytes. Representative picture of hemagglutination assay results is shown. Mouse erythrocyte suspension was mixed with viral stocks and incubated at room temperature for 1 h. Influenza A H3N2 reported to hemagglutinate erythrocytes [36] and HCoV-NL63 which does not bind to sialic acids (SAs) [33] were used as control samples.

**Figure 3:**
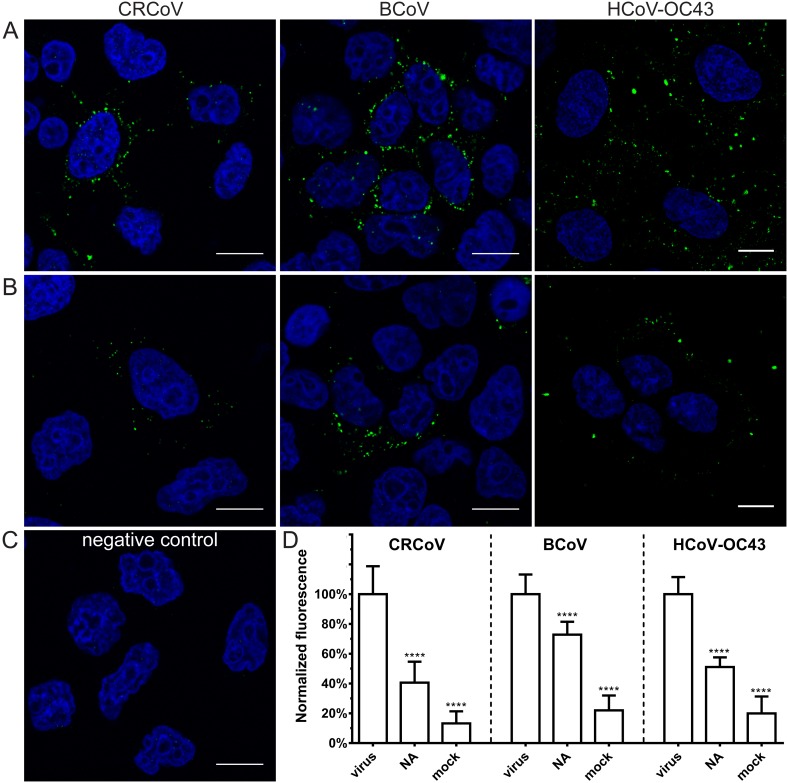
Removal of SAs from the surface of the cell limits attachment of HCoV-OC43 VR-1558, BCoV, and CRCoV to different extent. Cells pretreated with type II neuraminidase (NA, 200 mU/mL) were overlaid with HCoV-OC43, BCoV, and CRCoV stocks, incubated for 2 h at 4 °C, fixed, and immunostained. Viral capsids are presented in green, while blue denotes DNA. Scale bar 10 µm. Data were collected from a minimum of 12 fields of view, from at least three different samples. (**A**) virus inoculated, control cells; (**B**) virus inoculated, NA treated cells; (**C**) mock inoculated, non-treated cells; (**D**) FACS analysis of viral attachment in the presence of NA. Graph shows mean fluorescence normalized to control. The data is presented as mean ± SD from at least three experiments in triplicate. (* *p* < 0.05; ** *p* < 0.01; *** *p* < 0.001; **** *p* < 0.0001).

A number of betacoronaviruses, including HCoV-OC43 and BCoV, are thought to use SAs as entry receptors [9,10,11]. Our previous observations suggest that the role(s) of these molecules may be different from that reported in the literature; therefore, we performed a series of experiments to examine receptor usage. First, we performed a hemagglutination assay to verify whether these two viruses, and closely related CRCoV interact with these moieties. As shown in Figure 2, all three viruses agglutinated mouse erythrocytes, which are rich in SAs. Next, to determine the importance of SAs for CRCoV, BCoV, and HCoV-OC43 VR-1558 attachment, we treated cells with neuraminidase (NA) (to remove SA residues) prior to infection and then examined viral attachment. Removing SAs reduced attachment of all three viruses, with the strongest effect observed for CRCoV and HCoV-OC43 VR-1558 (59.4 ± 14.0% and 48.9 ± 6.5 decrease, respectively; Figure 3).

### 3.2. SAs Did Not Facilitate Entry of CRCoV, BCoV, and HCoV-OC43 VR-1558 to HRT-18G Cells

**Figure 4:**
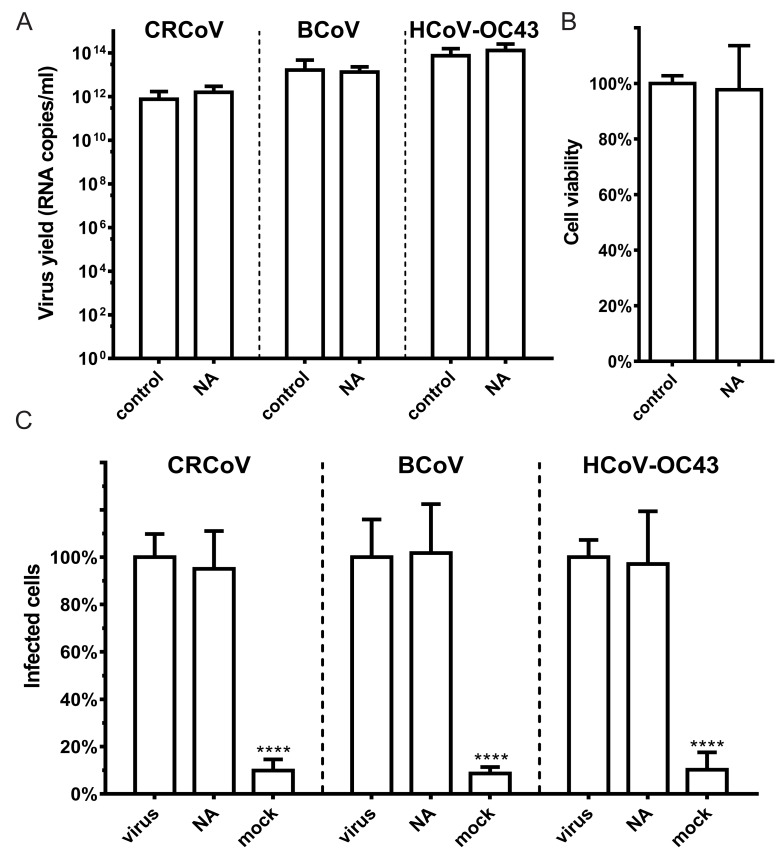
SAs removal does not affect HCoV-OC43 VR-1558, BCoV, or CRCoV replication. Cells pretreated with type II neuraminidase (NA, 200 mU/mL) were overlaid with HCoV-OC43 VR-1558, BCoV and CRCoV stocks (at TCID_50_ of 400 per milliliter, which approximately corresponds to M.O.I. = 0.0007) and incubated for 2 h at optimal temperature (see *Viral stocks*). Subsequently unbound virions were washed off and the cells were further incubated at optimal temperature for five days. (**A**) virus yield assessed by RT-qPCR. (**B**) NA effect on cell viability, as determined by an XTT assay. (**C**) The proportion of infected cells in the whole population normalized to control (flow cytometry). All data is presented as mean ± SD from at least three experiments in triplicate. (* *p* < 0.05; ** *p* < 0.01; *** *p* < 0.001; **** *p* < 0.0001).

To determine the role of SAs during CRCoV, BCoV, and HCoV-OC43 entry, we treated cells with neuraminidase (NA) prior to infection and then examined viral replication. Removal of SAs with NA had no effect on viral replication (Figure 4).

**Figure 5:**
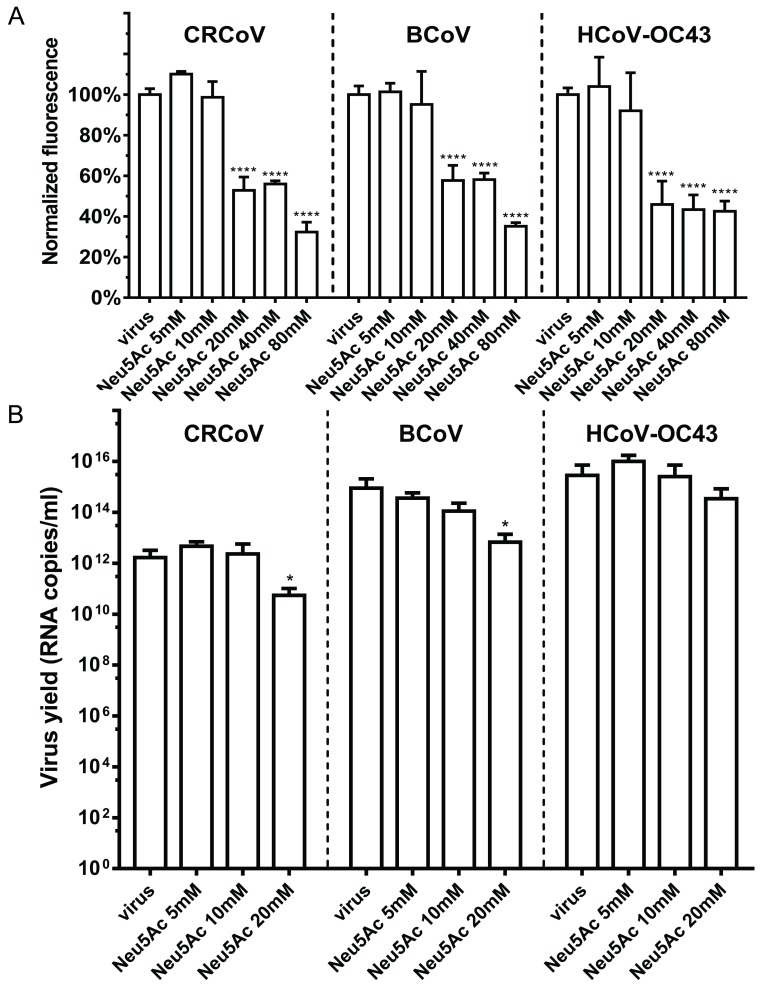
Soluble SAs limits attachment of HCoV-OC43 VR-1558, BCoV, and CRCoV but does not affect their replication. (**A**) Cells were overlaid with HCoV-OC43 VR-1558, BCoV, and CRCoV stocks preincubated with *N*-acetylneuraminic acid (Neu5Ac), incubated for 2 h at 4 °C, fixed, and immunostained. Graph shows results of flow cytometry analysis of viral attachment; data are presented as mean fluorescence normalized to control. (**B**) Cells overlaid with viral stocks (at TCID_50_ of 400 per milliliter, which approximately corresponds to M.O.I. = 0.0007) exposed to Neu5Ac were incubated for 2 h at temperature optimal for particular virus. Subsequently, unbound virions were washed off and cells were incubated for 5 days in the presence of Neu5Ac. The data is presented as mean ± SD of virus yield in cell culture supernatant (RT-qPCR) from at least three experiments in triplicate. (* *p* < 0.05; ** *p* < 0.01; *** *p* < 0.001; **** *p* < 0.0001).

To ensure that obtained results are valid and that reconstitution of the SAs on cell surface or incomplete scission of the SAs by NA does not affect the outcome, the infection was carried out in the presence of soluble SAs (Neu5Ac) prior to infection. The role of SAs during virus attachment and lack of effect on replication was confirmed for all three viruses (Figure 5).

### 3.3. Restoration of SAs on the Cell Surface Rescues Attachment of CRCoV, BCoV, and HCoV-OC43 VR-1558

**Figure 6:**
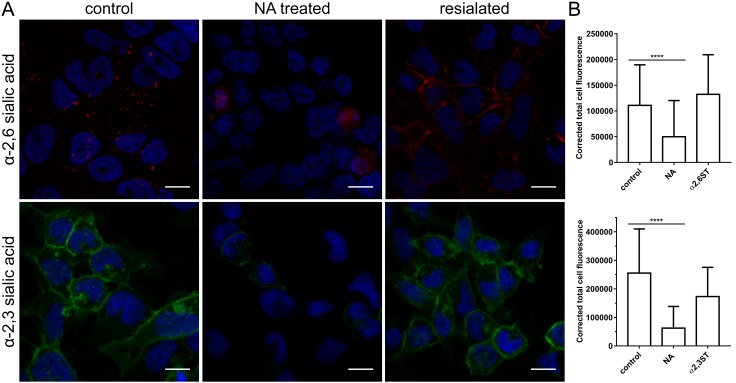
Activity of neuraminidase and sialyltransferases. (**A**) HRT-18G cells were treated with type II neuraminidase (NA, 500 mU/mL) and overlaid with α-2,3-sialyltransferase (α2,3-ST) or α-2,6-sialyltransferase (α2,6-ST) in the presence of 1 mM cytidine-5′-monophospho-*N*-acetylneuraminic acid for 2 h at 37 °C. Following treatment, cells were fixed, and SAs were visualized with *sambucus nigra* lectin (α-2,3-SAs specific) labelled with fluorescein (in green) or *maackia amurensis* lectin (α-2,6-SAs specific) labelled with Cy3 (in red). Scale bar 10 µm. Data were collected from a minimum of 12 fields of view, from at least three different samples. (**B**) Graphs present corrected total cell fluorescence calculated by subtracting the product of multiplying the cell surface and the mean background fluorescence from total cell fluorescence. The data is presented as mean ± SD from at least one hundred cells per condition. (* *p* < 0.05; ** *p* < 0.01; *** *p* < 0.001; **** *p* < 0.0001).

To confirm that the observed effect was SA-specific, we stripped and then restored SAs to the cell surface. First, HRT-18G cells were treated with NA to remove SAs. Then, the cells were incubated for 2 h at 37 °C with α-2,3-sialyltransferase (α2,3-ST) or α-2,6-sialyltransferase (α2,6-ST) in the presence of 1 mM of cytidine-5′-monophospho-N-acetylneuraminic acid sodium salt (CMP-Neu5Ac). Compounds’ activity was verified using α-2,3-SAs and α-2,6-SAs specific lectins (Figure 6).

**Figure 7:**
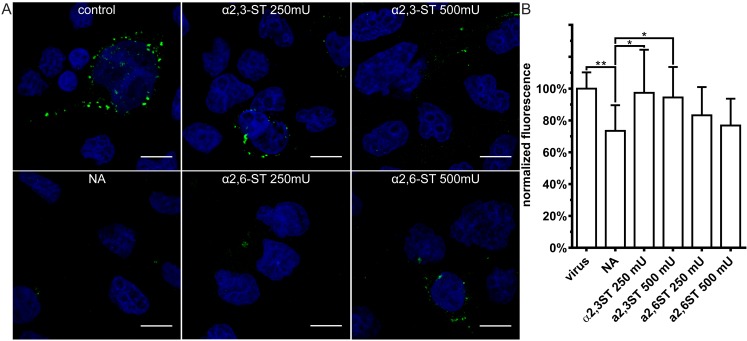
Resialylation restores CRCoV attachment to SAs-depleted cells. Cells treated with type II neuraminidase (NA, 500 mU/mL) and overlaid with α-2,3-sialyltransferase (α2,3-ST) or α-2,6-sialyltransferase (α2,6-ST) in the presence of 1 mM cytidine-5′-monophospho-*N*-acetylneuraminic acid for 2 h at 37 °C. Next, cells were overlaid with iodixanol-concentrated CRCoV, incubated for 2 h at 4 °C, fixed and immunostained. (**A**) Confocal analysis of CRCoV attachment in presence of NA and STs. Virions are presented in green, while blue denotes DNA. Scale bar 10 µm. Data were collected from a minimum of 12 fields of view, from at least two different samples. (**B**) FACS analysis of viral attachment in presence of NA and STs. Graph shows median fluorescence normalized to control. All data is presented as mean ± SD from at least three experiments in triplicates. (* *p* < 0.05; ** *p* < 0.01; *** *p* < 0.001; **** *p* < 0.0001).

**Figure 8:**
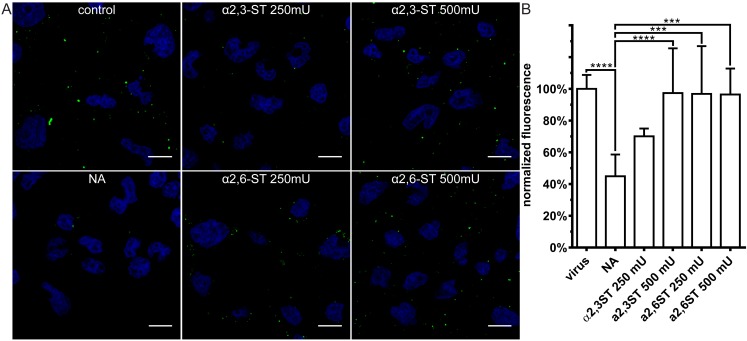
Resialylation restores HCoV-OC43 VR-1558 attachment to SAs-depleted cells. Cells treated with type II neuraminidase (NA, 500 mU/mL) and overlaid with α-2,3-sialyltransferase (α2,3-ST) or α-2,6-sialyltransferase (α2,6-ST) in the presence of 1 mM cytidine-5′-monophospho-*N*-acetylneuraminic acid for 2 h at 37 °C. Next, cells were overlaid with iodixanol-concentrated HCoV-OC43 VR-1558, incubated for 2 h at 4 °C, fixed, and immunostained. (**A**) Confocal analysis of HCoV-OC43 attachment in presence of NA and STs. Virions are presented in green, while blue denotes DNA. Scale bar 10 µm. Data were collected from a minimum of 12 fields of view, from at least two different samples. (**B**) FACS analysis of viral attachment in presence of NA and STs. Graph shows median fluorescence normalized to control. All data is presented as mean ± SD from at least three experiments in triplicates. (* *p* < 0.05; ** *p* < 0.01; *** *p* < 0.001; **** *p* < 0.0001).

**Figure 9:**
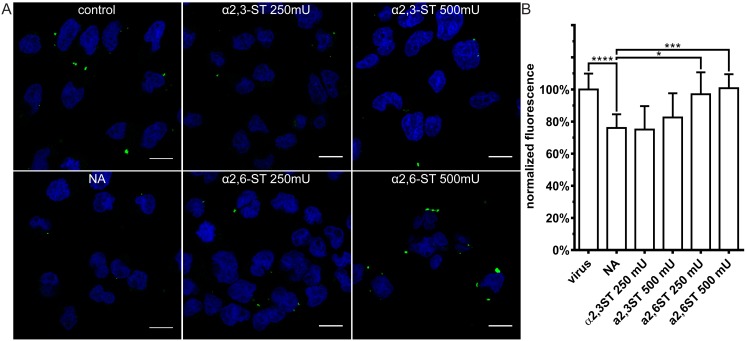
Resialylation restores BCoV attachment to SAs-depleted cells. Cells treated with type II neuraminidase (NA, 500 mU/mL) and overlaid with α-2,3-sialyltransferase (α2,3-ST) or α-2,6-sialyltransferase (α2,6-ST) in the presence of 1 mM cytidine-5′-monophospho-*N*-acetylneuraminic acid for 2 h at 37°C. Next, cells were overlaid with iodixanol-concentrated BCoV, incubated for 2 h at 4 °C, fixed and immunostained. (**A**) Confocal analysis of BCoV attachment in presence of NA and STs. Virions are presented in green, while blue denotes DNA. Scale bar 10 µm. Data were collected from a minimum of 12 fields of view, from at least two different samples. (**B**) FACS analysis of viral attachment in presence of NA and STs. Graph shows median fluorescence normalized to control. All data is presented as mean ± SD from at least three experiments in triplicates. (* *p* < 0.05; ** *p* < 0.01; *** *p* < 0.001; **** *p* < 0.0001).

We then examined virus attachment to control cells, to cells stripped of SA, and to cells on which the SA shield was restored. Restoration of the SAs rescued cell surface binding by all viruses (Figure 7, Figure 8 and Figure 9). CRCoV showed a clear preference for α2,3-SA moieties (Figure 7), while HCoV-OC43 bound preferentially to α2,6-linked moieties (Figure 8). Similar to HCoV-OC43 VR-1558, BCoV bound preferentially to α2,6-SA, but to much lower extent (Figure 9).

These results confirm that all three viruses require SAs for attachment: CRCoV shows a preference for α2,3-SA, BCoV for α2,6-SA, and HCoV-OC43 VR-1558 for α2,6-SA as previously reported for MDCK I cell line [37].

### 3.4. Interaction with HLA-I Molecules

**Figure 10:**
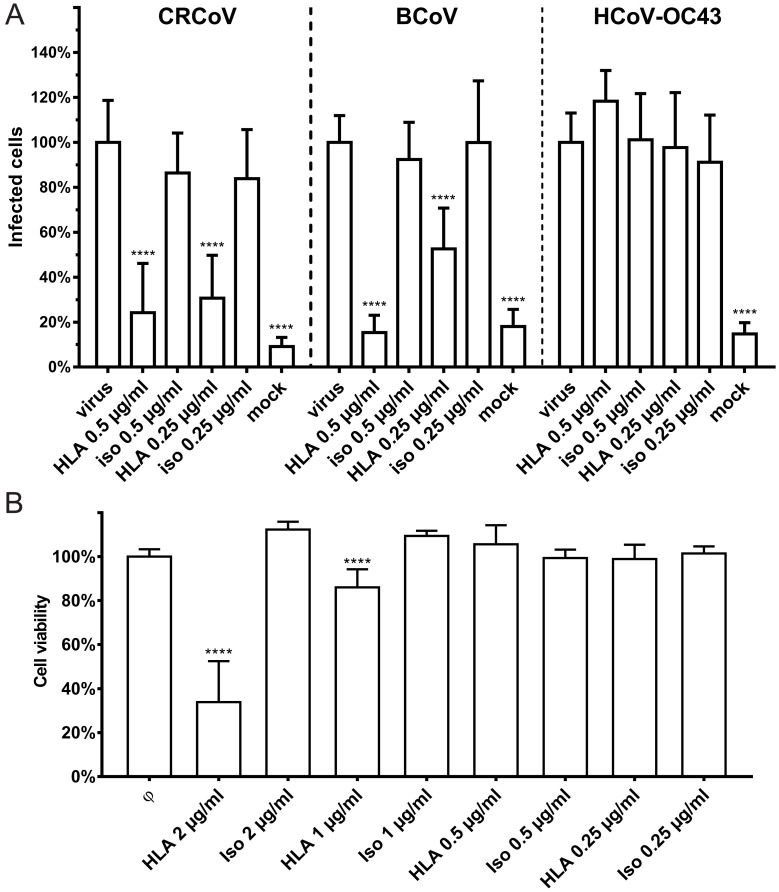
Human leukocyte antigen (HLA) class I as entry receptor for betacoronaviruses. Cells infected with HCoV-OC43 VR-1558, BCoV and CRCoV (at TCID_50_ of 400 per milliliter, which approximately corresponds to M.O.I. = 0.0007) in the presence of HLA class I antibodies were incubated for five days at optimal temperature. (**A**) The proportion of virus infected cells determined with flow cytometry normalized to control. (**B**) Cytotoxicity of antibodies determined with XTT assay. All data is presented as mean ± SD. (* *p* < 0.05; ** *p* < 0.01; *** *p* < 0.001; **** *p* < 0.0001).

As we discovered that SAs do not serve as entry receptors, we examined some other candidates. One protein reported to facilitate entry of HCoV-OC43 and HCoV-HKU1 is the HLA molecule. Therefore, we blocked the interaction between the virus and HLA-I molecules using polyclonal antibodies. This had no effect on replication of HCoV-OC43 VR-1558. However, it did block infection by CRCoV and BCoV, suggesting that HLA-I serves as an entry receptor for these viruses (Figure 10).

### 3.5. Involvement of HS and Lectins

**Figure 11:**
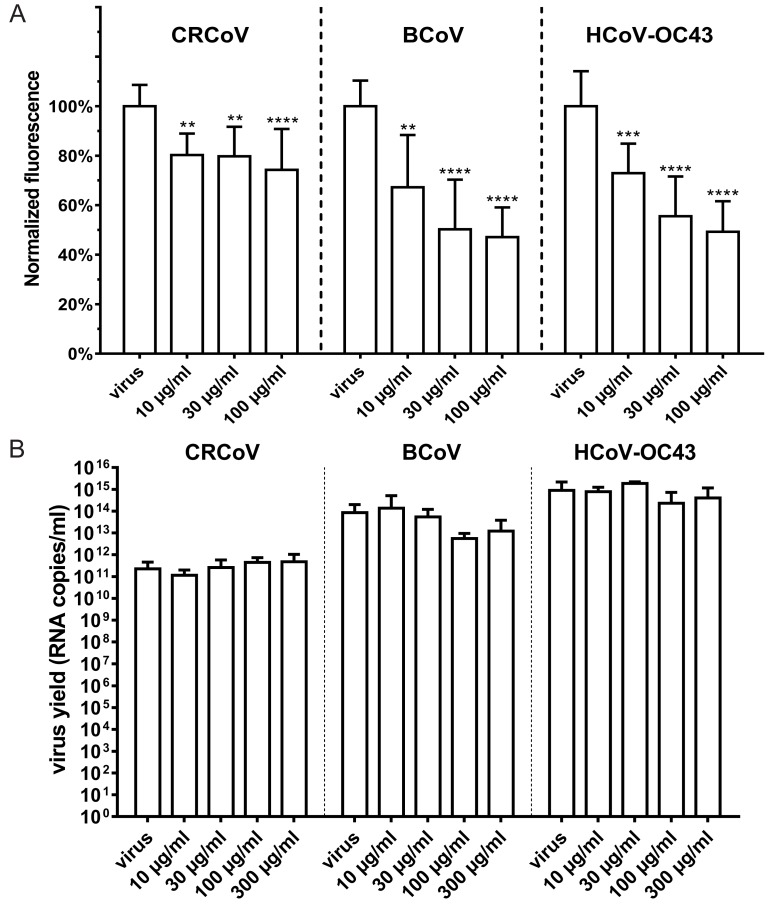
Heparan sulfate (HS) as attachment receptor for HCoV-OC43 VR-1558, BCoV, and CRCoV. (**A**) FACS analysis of viral attachment in the presence of HS. Cells were overlaid with HCoV-OC43 VR-1558, BCoV, and CRCoV stocks in the presence of HS, incubated for 2 h at 4 °C, fixed, and immunostained. Graph shows mean fluorescence normalized to control. (**B**) Analysis of viral entry in the presence of HS. Cells were overlaid with HCoV-OC43, BCoV, and CRCoV stocks (at TCID_50_ of 400 per milliliter, which approximately corresponds to M.O.I. = 0.0007) in the presence of HS and incubated for 2 h at optimal temperature. Subsequently unbound virions were washed off and the cells were further incubated at optimal temperature in the presence of HS. Virus yield was assessed by RT-qPCR at 5th day p.i. All data is presented as mean ± SD from at least three experiments in triplicate. (* *p* < 0.05; ** *p* < 0.01; *** *p* < 0.001; **** *p* < 0.0001).

Even though removal of SAs from the cell surface resulted in potent inhibition of viral attachment to the cell surface, the effect was not complete. Therefore, we examined the role(s) of HS and other sugar moieties (d-(+)-galactose, d-(+)-glucose, d-(+)-mannose, or *N*-acetyl-d-glucosamine) on virus attachment and entry. Only HS had a marked effect on CRCoV, BCoV, and HCoV-OC43. Inhibition was most pronounced for BCoV (49.8 ± 20.1%; 30 µg/mL HS) and HCoV-OC43 (44.4 ± 16.0%; 30 µg/mL HS), and to the lesser extent for CRCoV (25.7 ± 16.5%; 100 µg/mL HS) (Figure 11A). There was no significant effect on replication of any of the viruses (Figure 11B). Among the sugars tested, NAc alone showed a slight inhibitory effect on HCoV-OC43 VR-1558 attachment but had no effect on that of CRCoV or BCoV (data not shown [38]).

### 3.6. Attachment Receptors and Adaptation during Cell Culture

**Figure 12:**
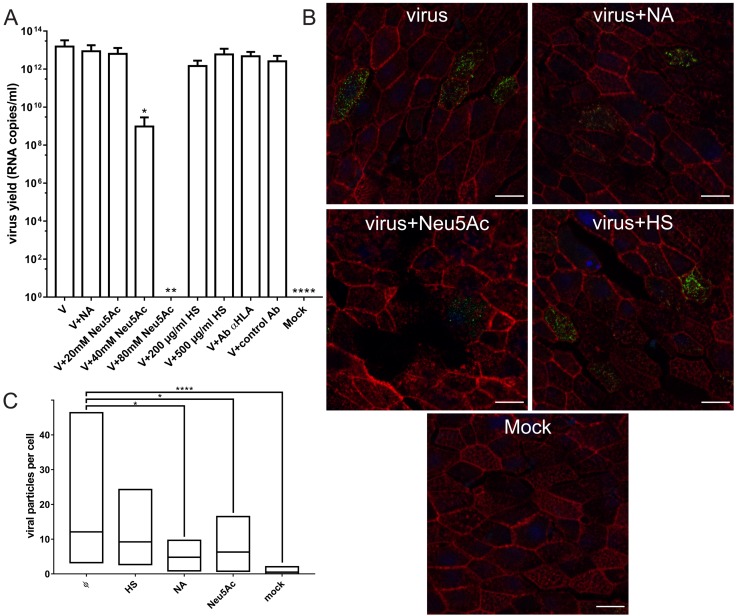
Attachment and replication of HCoV-OC43 0500 in human airway epithelium (HAE) cultures. (**A**) SAs serve as entry receptors for HCoV-OC43 0500. Influence of NA (200 mU/mL), HS (200 µg/mL), Neu5Ac (20–80 mM), and HLA class I specific antibodies (0.5 µg/mL) on HCoV-OC43 0500 infection. Viral yield was assessed by RT-qPCR 5 days p.i. The data is presented as mean ± SD from at least two experiments in duplicates. (* *p* < 0.05; ** *p* < 0.01; *** *p* < 0.001; **** *p* < 0.0001). (**B**) SAs serve as attachment receptors for HCoV-OC43 0500. Confocal analysis of NA (200 mU/mL), Neu5Ac (40–80 mM) and HS (200 µg/mL) effect on HCoV-OC43 0500 attachment. Viral capsids are presented in green, blue denotes DNA and red represents actin. Scale bar 10 µm. (**C**) Quantification of virus attachment. A number of viral particles that attached to the surface of fully differentiated HAE culture was graphed. Data were collected from a minimum of 12 fields of view, from at least two different samples. Number of particles and number of cells were quantified using ImageJ Fiji built in tool “3D Objects Counter”. Results are presented as min-max graph with line corresponding to the mean value (* *p* < 0.05; ** *p* < 0.01; *** *p* < 0.001; **** *p* < 0.0001).

To test the possibility that the observed specificity of HCoV-OC43 resulted from adaptation during cell culture, we examined differences in cell attachment and entry for laboratory and clinical strains of HCoV-OC43. It is worth remembering that the laboratory strain has lost the ability to infect human airway epithelium [39], while the clinical strain does not replicate in cell lines. We performed a series of experiments in which HCoV-OC43 0500 (the clinical strain) was used to infect HAE cultures. Neither HS competition, nor specific blockade of HLA-I or sugar binding moieties affected viral replication, suggesting that these molecules do not play a role in cell entry by HCoV-OC43 0500. However, supplementation with soluble SAs resulted in dose-dependent reduction in replication, implying SAs role in entry process also for the clinical strain (Figure 12A).

Furthermore we found that only SAs but not HS had effect on HCoV-OC43 0500 adhesion to HAE cultures (Figure 12B,C).

## 4. Discussion

To date, studies on betacoronavirus entry show high variability in terms of receptors used for cell attachment and entry [9,10,11,12,13,15,16,17]. To make an example, SARS-CoV uses angiotensin-converting enzyme 2 (ACE2), a type I integral transmembrane protein as entry receptor [40,41], but it may also utilize c-type lectin receptor expressed by dendritic cells (DC-SIGN), a DC-SIGN-related molecule (L-SIGN), and vimentin [42,43,44,45,46,47]. On the other hand, MERS-CoV, which also infects human respiratory tract epithelium, employs dipeptidyl peptidase 4 to enter the cell [48]. Other, nonhuman betacoronaviruses exploit diverse molecules, such as CEACAM1 (MHV), neural cell adhesion molecule (porcine hemagglutinating encephalomyelitis coronavirus) [49], or SAs (HCoV-OC43) [37].

HCoV-OC43 is believed to use SAs as attachment and entry receptors, but it was also reported to employ HLA-I to enter the cell [15,16]. The BCoV has been extensively studied in the context of SAs usage. Models based on chicken, mouse, and rat erythrocytes (hemagglutination) and MDCK I, CaCo-2, and LLC-PK1 cell lines (entry and replication) confirmed the role of SAs in attachment of BCoV, but no information on the role of these moieties for the cell entry has been available [9,10,11,12,37,50]. CRCoV receptors remain unknown.

Here, we checked whether HCoV-OC43, CRCoV, and BCoV interact with SAs. First, the hemagglutination assay yielded positive results in all the cases; we made an effort to establish the exact role of these moieties during virus–cell interaction. In all three cases, depletion of SAs from target cells as well as blockade of virus and SAs interaction using soluble neuraminic acid had a strong effect on virus binding, proving that these moieties serve as attachment receptors. Reconstitution of the SAs on cell surface after enzymatic removal revealed that CRCoV binds preferentially to α-2,3-linked SAs but not to α-2,6-linked SAs. By contrast, HCoV-OC43 VR-1558 used α-2,6-SAs rather than α-2,3-SAs, consistently with the published literature [37]. Although the preference of BCoV was similar to that of HCoV-OC43, it was less pronounced, suggesting a possible role of other molecules in the virus attachment to HRT-18G cells.

Apart from N-acetylneuraminic acid (Neu5Ac), N-glycolylneuraminic acid (Neu5Gc) is also present on most mammalian cells surface [27,51,52,53]. Neu5Gc is a derivative of Neu5Ac as a consequence of the activity of citidine monophosphate N-acetylneuraminic acid hydroxylase (CMAH). Due to deletion in CMAH gene, human cells are deprived of Neu5Gc [53]. Although it is commonly believed that Neu5Gc prevails in nonhuman animal cells, currently available literature shows that dog ocular mucins and erythrocytes of most western breeds are rich in Neu5Ac rather than Neu5Gc [52,54,55]. Besides it is also known that bovines express both Neu5Ac and Neu5Gc in their tissues [51,53].

As α-2,6-SAs dominate in human respiratory tract [56,57], the HCoV-OC43 preference for them is not surprising. It is worth stressing that α-2,3-SAs are abundant on ciliated epithelial cells [56,57], which is in line with CRCoV role in CIRD development, as disruption of ciliary clearance is one of the early hallmarks of the disease [27]. Of interest, Bakkers et al. [58] recently reported loss of hemagglutinin-esterase (HE) mediated SAs binding for HCoV-OC43 and HCoV-HKU1 during adaptation to human tissue, which suggests that the observed attachment is mediated by the spike protein.

The role of SAs in adhesion of these three betacoronaviruses is unquestionable, but we made an effort to check whether other moieties may also serve as attachment receptors for the viruses, as blockade of virus–SAs interaction in some cases inhibited virus attachment pronouncedly, but incompletely. Of the tested ones, soluble HS hampered attachment of all three betacoronaviruses but did not severely affect their replication. The most pronounced effect was noted for HCoV-OC43 and the weakest for CRCoV. Affinity for HS has been previously described to emerge during adaptation to cell culture [59,60]; therefore, one may assume that the observed effect would not be valid for a wild-type virus. As we had a unique opportunity to test this hypothesis on HCoV-OC43 0500 virus (which does not replicate in vitro), we used fully differentiated HAE cultures and a standard cell line to show that attachment of the clinical strain does not depend markedly on HS, and that the ability to bind to HS most likely is related to the cell culture adaptation [61]. HRT-18G cells constitute the only existing model for the CRCoV infection in vitro and we decided to study CRCoV and BCoV in vitro on these cells [62]. Consequently, one cannot rule out the possibility that attachment receptor specificity may also be altered due to the cell culture adaptation. While SAs appeared to be important for virus binding to the cell surface, they are not essential for entry of HCoV-OC43 VR-1558, CRCoV, and BCoV. Importantly, the clinical strain of HCoV-OC43 0500 appear to employ this molecule as an adhesion and entry receptor, as soluble SAs block the attachment and entry of the virus to the human airway epithelium. Depletion of SAs with neuraminidase only mildly affected virus attachment and entry, but we believe that this is due to incomplete removal of SAs, the ability of cells to reconstitute SAs on the surface and the experimental settings.

Because the results showed that SAs do not serve as entry receptors for HCoV-OC43 VR-1558, CRCoV, or BCoV, we aimed to identify the molecules that facilitate cell entry by these viruses. Studies conducted in the mid-1990s showed that HLA served as an HCoV-OC43 entry receptor on RD and HCN-lA cells [15,16,63]; more recent studies show that it also acts as an entry receptor for HCoV-HKU1 [17]. These findings, however, have not been confirmed by other research groups until now [64]. Here, we examined the role of HLA-I as a receptor for all three betacoronaviruses and found out that it does not play a role in cell entry by the laboratory and clinical strains of HCoV-OC43. However, replication of BCoV and CRCoV was hampered in the presence of HLA-specific antibodies, showing that these viruses employ HLA-I as entry receptors. HLA class I molecules structurally consist of three α chains linked with a β_2_-microglobulin molecule. The membrane proximal end is formed by the α_3_ and β_2_M domains, which adopt a standard immunoglobulin-like fold [65]. Consequently, they belong to the immunoglobulin superfamily. Interestingly, this superfamily also includes CAECAM1, which serves as a receptor for MHV [16,17]. It will not be too farfetched a presumption to assume that cousins of MHV—CRCoV and BCoV—use structurally similar molecules as their entry receptors.

Despite similarities between betacoronaviruses on the level of protein sequence and the range of surface molecules recognized by them, the prevailing opinion is that they form separate genera characterized by very narrow host ranges [59]. However, a recent report regarding HCoV-OC43 outbreak among wild chimpanzees suggests that betacoronaviruses may undergo recurrent interspecies transmissions [66]. Considering previous reports, one may conclude that these three betacoronaviruses frequently cross species boundaries, leading to recombination and emergence of intermediate forms [27,67,68,69]. As all three viruses can replicate in human cells, this scenario is realistic; however, epidemiological studies are needed to confirm the hypothesis. One may also speculate that separation of BCoV and OC43 has not really occurred, and that some lineages belonging to a single species are able to infect a number of different hosts. On the other hand, it is also possible that HLA-I specificity is related to the cell culture adaptation.

To summarize, we show here that three closely related betacoronaviruses (HCoV-OC43 VR-1558, BCoV, and CRCoV) utilize SAs and HS to attach to the cell surface of target cells. However, they show different preferences for these moieties, and the importance of these interactions is debatable. HLA-I molecules serve as entry receptors for CRCoV and BCoV, but not for HCoV-OC43 0500 for which SAs carry both roles.

## Figures and Tables

**Table 1:** Probes and primers used in polymerase chain reaction (PCR) assay.

Virus	Probe Sequence	Fluorescent Dyes	Forward Primer Sequence (5’→3’)	Reverse Primer Sequence (5’ → 3’)	Amplification Program	PCR Product Sequence
HCoV-OC43	TGACATTGTCGATCGGGACCCAAGTA	FAM (6- carboxyfluorescein) and TAMRA (6- carboxytetramethyl-rhodamine)	AGCAACCAGGCTGATGTCAATACC	AGCAGACCTTCCTGAGCCTTCAAT	50 °C for 2 min, 92 °C for 10 min, 40 cycles of 92 °C for 15 s and 60 °C for 1 min	AGCAACCAGGCTGATGTCAATACCCCGGCTGACATTGTCGATCGGGACCCAAGTAGCGATGAGGCTATTCCGACTAGGTTTCCGCCTGGCACGGTACTCCCTCAGGGTTACTATATTGAAGGCTCAGGAAGGTCTGCT
CRCoV and BCoV	AGATCTACTTCACGCGCATCCAGT	FAM and TAMRA	CAGGAAGGTCTGCTCCTAATTC	GTTGCCAGAATTGGCTCTACTA	95 °C for 5 min, 30 cycles of 95 °C for 15 s and 60 °C for 30 s	CAGGAAGGTCTGCTCCTAATTCCAGATCTACTTCACGCGCATCCAGTAGAGCCTCTAGTGCAGGATCGCGTAGTAGAGCCAATTCTGGCAAC

## Supplementary Materials

The following are available online at https://www.mdpi.com/1999-4915/11/4/328/s1, Figure S1: FACS analysis, Figure S2: Replication of selected betacoronaviruses.
