# Supplementary material for: Canine Respiratory Coronavirus, Bovine Coronavirus, and Human Coronavirus OC43: Receptors and Attachment Factors
Source: Viruses. 2019 Apr 5;11(4):328. doi: 10.3390/v11040328 (PMC6521053; doi:10.3390/v11040328)
Supplement: Supplementary file 1 [file viruses-11-00328-s001.pdf]

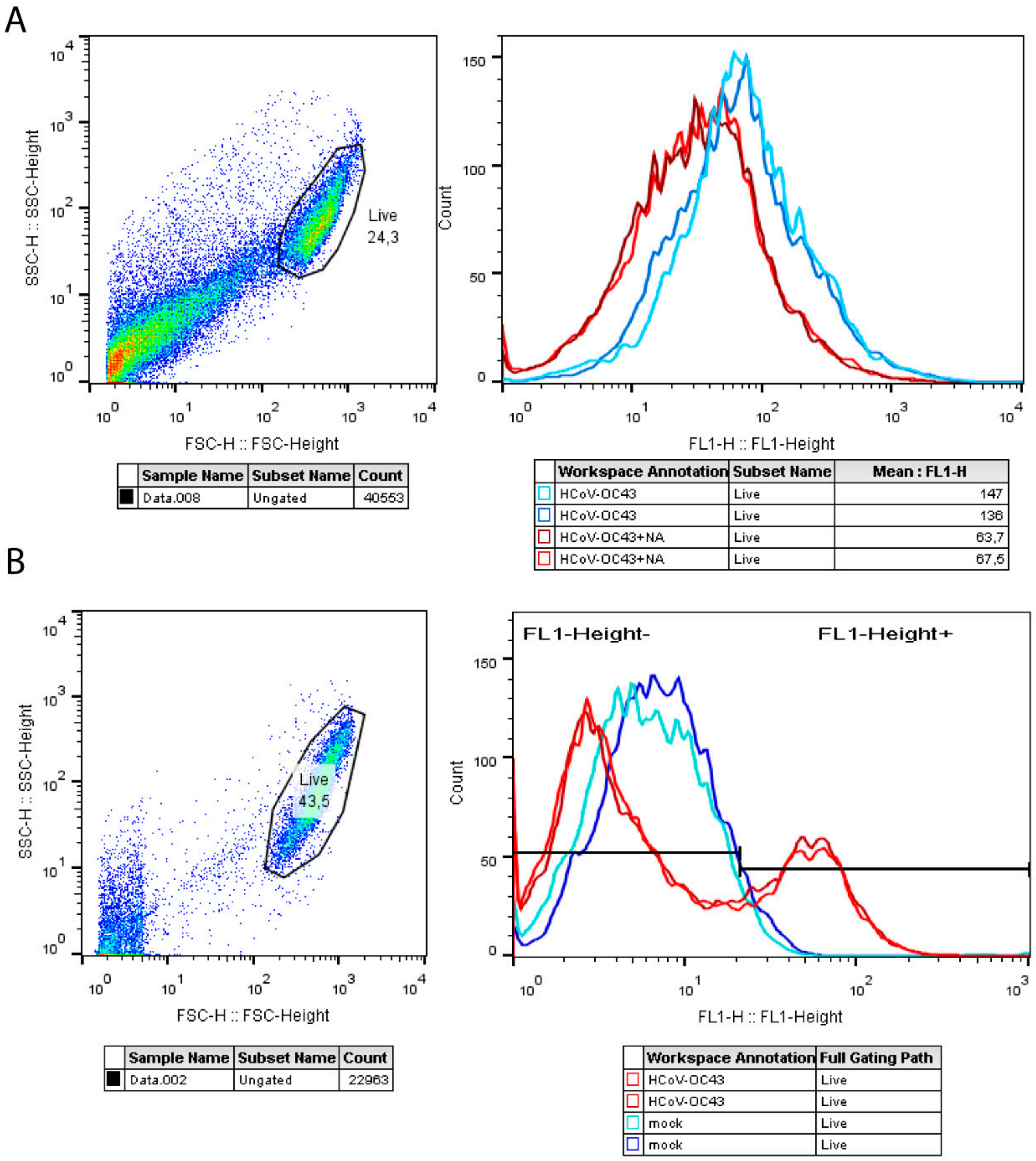

Figure S1. FACS analysis. (**A**) FACS analysis of HCoV-OC43 attachment to HRT-18G cells pretreated with neuraminidase. Upon experimental procedure, fixed cells were permeabilized and immunostained with antibodies specific to viral nucleocapsid proteins labeled with Alexa Fluor 488. Cells were gated as indicated in FSC/SSC graphs to select for an intact cell population. Fluorescence intensity for this cell population corresponding to the N protein quantity was assessed and is presented in FL1-H histograms. Red lines: virus-inoculated cells pretreated with neuraminidase; blue lines: virus-inoculated control cells. (**B**) FACS analysis of HCoV-OC43 replication in HRT-18G cells. Infected cells were trypsynized and fixed at day 5 p.i., permeabilized and immunostained with antibodies specific to viral nucleocapsid proteins labeled with Alexa Fluor 488. Fluorescence intensity for this cell population corresponding to the N protein quantity was assessed and is presented in FL1-H histograms. Red lines: virus-infected cells; blue lines: mock-inoculated cells.

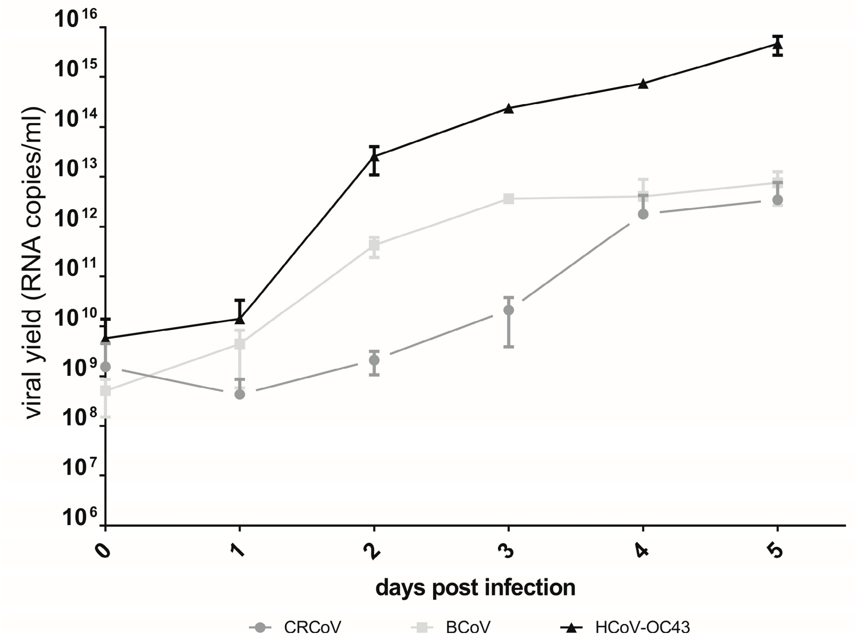

Figure S2. Replication of selected betacoronaviruses. HRT-18G cells were infected with HCoV-OC43, BCoV, or CRCoV stocks ($TCID_{50}$ of 400 per milliliter, M.O.I. of ~0.0007) and incubated for 2 h. Next, inoculum was discarded, cultures were thoroughly washed and samples for analysis were collected every 24 h. Virus yield was assessed by RT-qPCR. All data is presented as mean ± SD
